# Communication-related aspects of prospective memory: an exploratory factor analysis of prospective memory questionnaires

**DOI:** 10.1590/2317-1782/20242023233en

**Published:** 2024-05-20

**Authors:** Dasmine Fraclita D’Souza, Gagan Bajaj, Himani Kotian, Sheetal Raj Moolambally, Jayashree S. Bhat

**Affiliations:** 1 Department of Audiology and Speech Language Pathology, Kasturba Medical College Mangalore, Manipal Academy of Higher Education - Manipal, Karnataka, India.; 2 Department of Community Medicine, Kasturba Medical College Mangalore, Manipal Academy of Higher Education - Manipal, Karnataka, India.; 3 Department of Medicine, Kasturba Medical College Mangalore, Manipal Academy of Higher Education - Manipal, Karnataka, India.; 4 Nitte Institute of Speech and Hearing - Mangalore, Karnataka, India.

**Keywords:** Prospective Memory, Self-perception, Questionnaire, Communication, Speech Language, Pathology

## Abstract

**Purpose:**

Prospective memory (PM) questionnaires are frequently used to evaluate perceptions of PM skills in daily life. This study aimed to systematically investigate communication-specific attributes using pre-existing PM self-rating questionnaires to inform clinicians and researchers about the role of PM in cognitive communicative evaluations.

**Methods:**

PM-related items from three questionnaires (i.e., Prospective Memory Questionnaire, Comprehensive Assessment of Prospective Memory, and Prospective and Retrospective Memory Questionnaire) were compiled and embedded in Google Forms and distributed to 70 Speech-Language Pathologists (SLPs) with expertise in Cognitive Communicative Disorders across India. Participants first identified items related to communication, and were then contacted to rate the communication-related PM items using a Likert scale for their degree of appropriateness. Responses from 40 SLPs were obtained and subjected to item-content validity index (i-CVI) and exploratory factor analysis (EFA).

**Results:**

Of the 114 PM items, 28 received ratings over 50% for their relevance to communication. Of the 28 items, 21 had an i-CVI score greater than 0.8. After the removal of overlapping content, 14 items were finalized and subjected to EFA, which resulted in four factors: PM failure due to loss of communicative content, PM failure due to loss of communicative intent, PM cost due to ongoing interference, and PM failure linked to the priority of communicative intent.

**Conclusion:**

This study highlights communication-related aspects of PM that can be used as a framework for SLPs to assess and research PM skills.

## INTRODUCTION

‘Thinking of something I must do but tend to forget to do it’, ‘Losing track of the things I need to do during the day’, ‘particularly the less common ones like forgetting to pay a bill’, ‘Going to the supermarket and forgetting to get what I went for’ are a few common prospective Memory-related complaints among aging adults^(1)^. Prospective Memory (PM) refers to an individual’s ability to execute pre-planned intentions, thoughts, or actions in the future^(2)^. PM processing is believed to occur across five stages: the formation of intention (creating the intention), retention interval (the period during which the intention is retained), performance interval (the period during which the intention is executed), initiation and execution of the intended action (the beginning and completion of the intended action), and outcome evaluation (evaluating whether the created intention is accomplished)^(3)^. Similar to other cognitive skills, PM declines with healthy aging. Previous studies have indicated that younger adults typically outperform older adults on laboratory tasks^(4)^. A decrease in PM has been observed in various clinical groups, including those with cognitive communicative disorders, such as mild cognitive impairment, dementia, traumatic brain injury, Parkinson’s disease, and autism spectrum disorder^(2)^.

When various elements of daily activities intertwine with regular cognitive processes, they can sometimes result in lapses in PM, significantly affecting an individual. While PM involves intending to do something, many daily activities tend to become habitual. However, when these routines are disrupted, they can cause inconvenience in daily life and potentially lead to serious consequences in both the professional^(5)^ and personal spheres^(6)^. For instance, a child tragically died of heatstroke because his father was preoccupied with work-related thoughts inadvertently overlooking the child in the backseat of his car. Similarly, in a professional setting, a newly hired faculty member experienced embarrassment when, despite reminders on his wall and desk calendar, he missed attending his first faculty meeting at a new college^(6)^.

PM significantly influences various aspects of daily communication. These include tasks such as remembering crucial details or information during conversations, responding to emails within specified timelines, making or returning calls promptly, attending important meetings or appointments, and ensuring the transmission of vital information to others. Robust PM abilities are crucial for adhering to social norms^(7)^, such as remembering to greet individuals, maintaining eye contact, or appropriately acknowledging conversations, thereby enhancing overall communication effectiveness. PM functions linked to communication play a pivotal role in fulfilling social obligations and improving interpersonal interactions^(7)^, both personally and professionally. Lapses in communication-related PM can lead to social challenges and impact one's credibility. Unlike retrospective memory, PM carries a moral aspect; failure in PM tasks is often perceived as unreliable, affecting one's reputation and sense of self, thereby emphasizing its critical social importance^(7)^.

Various cognitive constructs integral to communication, such as attention, perception, working memory, short-term memory, problem-solving, and reasoning, are routinely assessed by Speech-Language Pathologists (SLP)^(8)^. Despite the pivotal role of PM in communication functions, it has garnered less attention in cognitive communication assessment. Existing literature underscores PM tasks, such as the virtual week, used in cognitive communicative assessments for individuals with traumatic brain injury^(2)^. Several questionnaires, including the Prospective Memory Questionnaire (PMQ)^(9)^, Prospective and Retrospective Memory Questionnaire (PRMQ)^(10)^, Comprehensive Assessment of Prospective Memory (CAPM) questionnaire^(11)^, and Brief Assessment of Prospective Memory (BAPM)^(12)^, and Prospective Memory Concerns Questionnaire (PMCQ)^(13)^ have been used to assess PM abilities in clinical and non-clinical populations. However, these tools often lack specificity regarding the PM constructs related to everyday communication processes and deficits.

To emphasize the crucial role of PM in communication and its relevance to SLPs dealing with cognitive communicative assessment and intervention, the initial step would involve systematically identifying communication-related PM aspects. Consequently, this study aimed to extract communication-specific facets of PM from prevalent PM questionnaires. The objectives of this study were to identify the most appropriate communication-related PM items from existing PM questionnaires through expert ratings, followed by an analysis of the shortlisted items to categorize them according to the relevant constructs. We believe that a methodical, expert-driven identification of the specific attributes of communication-related PM could significantly aid SLP clinicians in conducting assessments and interventions for aging populations and individuals with cognitive communicative disorders. These identified attributes could serve as a crucial foundation for future SLP researchers, aiding in the development of targeted communication-specific PM assessment tools such as questionnaires and PM-based intervention strategies.

## METHODS

### Participants

The present study followed a cross-sectional research design, wherein the PM-related items of the selected PM questionnaires were presented to the study participants to identify the most appropriate communication-related PM aspects. Inspiration for the research design and method was drawn from a study conducted by Lemery et al.^(14)^. The research protocol was reviewed and approved by the Institutional Ethics Board (IEC KMC MLR 10-19/469). Prior to data collection, participants were presented with a statement of informed consent from Google Forms. Participants were required to indicate their consent to participate in the study by clicking on the 'I Agree' button before they could proceed.

Seventy actively employed and qualified SLPs were invited and 40 agreed to participate in the study. The participants in this study (mean age = 28.5±2.84 years) were post-graduates with at least one year of clinical experience (4.8±3.4 years) in treating individuals with cognitive communicative disorders. The demographic profiles of participants are presented in Table 1.

**Table 1:** Demographic description of the recruited Speech-Language Pathologists

Participant demographics	Speech-Language Pathologist
Gender	
Male	4
Female	36
	
Age	
Mean	28.53 years
SD	2.84 years
	
Work set up	
Academic	12
Hospital	10
Private practice	9
Freelance	1
Academic & Hospital	4
Private Practice & Hospital	2
Private Practice, Academic & Hospital	2

Caption: SD: Standard Deviation

### PM questionnaires

The selection of PM questionnaires for this study was guided by a recent systematic and meta-analytic review, as referenced by Blondelle^(15)^, which focused on instruments assessing PM abilities. The review scrutinized four specific questionnaires: the Prospective and Retrospective Memory Questionnaire (PRMQ), Comprehensive Assessment of Prospective Memory (CAPM), Prospective Memory Questionnaire (PMQ), and Brief Assessment of Prospective Memory (BAPM). They evaluated the instruments across ten critical criteria: translation, cross-cultural adaptation, validity, reliability, availability of normative data, type of PM assessed (event-based or time-based), diagnostic value, use of external aids, availability of parallel versions, qualitative scoring, and association with functional outcome measures. According to Blondelle^(15)^, three of the four questionnaires, that is, PRMQ, CAPM, and PMQ successfully met or exceeded at least 50% of these criteria. Consequently, they were specifically selected for inclusion in the present study.

#### Prospective and Retrospective Memory Questionnaires (PRMQ)

The PRMQ^(10)^ comprises 16 items inclusive of two domains that assess PM and retrospective memory, each of which contains eight items. The PM domain comprises four subscales, with two items each dedicated to prospective short-term self-cued, prospective short-term environmentally cued, prospective long-term self-cued, and prospective long-term environmentally cued. PRMQ is rated based on how often memory failure is observed in each domain on a 5-point Likert rating scale, wherein 1 indicates ‘Never,’ 2 indicates ‘Rarely,’ 3 indicates ‘Sometimes,’ 4 indicates ‘Quite often,’ and 5 indicates ‘Very often.’ Higher PRMQ scores indicate a higher frequency of memory failure. PM-related changes in both the typically aging population and those with Alzheimer’s dementia have been studied using the PRMQ^(16)^.

#### Prospective Memory Questionnaire (PMQ)

The self-rated PMQ^(9)^ includes four domains: long-term episodic scale, short-term habitual scale, internally cued scale, and techniques to assist with the memory scale. Each item is rated based on the frequency of PM memory failure on a visual analog scale ranging from 0 times or more, 4 times or more, or 6 times or more in a week, month, or year, respectively. Higher PMQ scores indicate more PM failures. The PMQ has been used to evaluate perceived PM deficits in patients with brain trauma and older adults^(9,17)^.

#### Comprehensive Assessment of Prospective Memory (CAPM)

CAPM^(11)^ comprises 54 items and three sections. Section A (CAPM/A) assesses the frequency of PM failures, Section B (CAPM/A) assesses the degree of concern about PM failure (with the same items as in Section A), and Section C (CAPM/A) assesses the reasons for PM failure. It comprises of a 5-point Likert Scale used to rate sections A and B, where 1 indicates ‘Never’; 2 indicates ‘Rarely’ (once/month); 3 indicates ‘Occasionally’ (2–3 times/month); 4 indicates ‘Often’ (once/week); and 5 indicates ‘Very often’ (daily). In Section C, each item is rated according to its agreement with its statements using a 4-point Likert scale, with 1 indicating strongly disagree, 2 indicating disagree, 3 indicating agree, and 4 indicating strongly agree. Higher scores indicate greater memory failure. Several researchers have used the CAPM to assess PM in aging populations^(18)^ and in those with brain damage^(19)^. Additionally, it has been used as a self-reported metric to complement virtual reality-based PM assessments^(19)^.

### Procedure

PM-related items from the three questionnaires were aggregated into a Google Form comprising 114 items shared with participants via a web link in personal emails. In the initial contact, participants were requested to assess each item on a binary scale, designating ‘1’ if the item pertained to communication, and ‘0’ otherwise. Items receiving over 50% ‘communication-related’ ratings were shortlisted for subsequent analysis.

The identified communication-related PM items were reintegrated into Google Forms and similarly redistributed in the second phase of communication. Participants were tasked with rating each item's appropriateness towards communication on a 5-point Likert scale: ‘1’ for ‘Not appropriate,’ ‘2’ for ‘Least appropriate,’ ‘3’ for ‘Can’t say,’ ‘4’ for ‘Somewhat appropriate,’ and ‘5’ for ‘Highly appropriate.’ Subsequently, an Item-Content Validity Index (i-CVI) analysis was conducted^(20)^. PM items scoring an i-CVI value exceeding 0.8 were selected for further scrutiny through exploratory factor analysis to unveil potential underlying patterns or themes associated with communication.

### Statistical analyses

Version 25 of IBM SPSS Statistics for Windows was used to analyze the data. Frequency distribution analysis was conducted to identify items relevant to communication. The i-CVI was computed based on ratings for appropriateness, indicating the proportion of experts identifying an item as either 'somewhat appropriate' or 'most appropriate' concerning communication^(20)^. Furthermore, we employed principal component analysis and varimax rotation in exploratory factor analysis to uncover potential underlying patterns or themes associated with communication.

## RESULTS

The frequency distribution analysis of responses following the initial participant correspondence revealed that 28 of the 114 items sourced from the three questionnaires obtained ratings exceeding 50%, indicating their association with communication. Subsequently, these 28 items underwent another round of participant ratings on a 5-point Likert scale to determine their appropriateness within the realm of communication and compute the i-CVI scores. Table 2 presents the frequency distribution of both the appropriateness ratings and i-CVI scores for each item.

**Table 2:** Frequency distribution of the appropriateness ratings from the 40 participants and i-CVI scores for each item

Questionnaire	Item description	Frequency of appropriateness ratings					i-CVI
		1	2	3	4	5	
PRMQ	“Do you decide to do something in a few minutes time and then forget to do it?”	1	2	6	11	20	0.8
	“Do you fail to mention or give something to a visitor that you were asked to pass on?”	2	2	5	9	22	0.8
	“If you tried to contact a friend or relative who was out, would you forget to try again later?”	1	4	7	9	19	0.7
	“Do you forget to tell someone something they had meant to mention a few minutes ago?”	1	6	2	11	20	0.8
CAPM	“Forgetting to pass on a message”	1	1	1	9	28	0.9
	“Forgetting to make a telephone call you intended to make”	3	0	3	11	23	0.9
	“Arriving at a shop and forgetting what you planned to buy”	1	1	3	12	23	0.9
	“Forgetting to mention a point you intended to make during a conversation”	1	1	5	12	21	0.8
	“Not remembering to pay bills”	4	0	2	16	18	0.9
	“Forgetting to meet a friend at the pre-arranged time”	2	2	3	9	24	0.8
	“When I forget to do something I had planned to do, it is usually because I forgot what I actually had to do”	4	1	6	7	22	0.7
	“I frequently forget to do things that other people have asked me to do”	1	3	5	8	23	0.8
	“I frequently forget to do things that I have planned to do”	2	5	5	8	20	0.7
	“I rely on other people to remind me when I have to remember to do things”	3	2	8	9	18	0.7
	“If I am engrossed in another task, I find it difficult to remember to do things”	2	1	6	14	17	0.8
	“Sometimes even though I remember that something has to be done, I forget to do it if I am interrupted (e.g., by a telephone call or by a person)”	3	0	7	12	18	0.8
PMQ	“I missed appointments I had scheduled”	5	4	1	9	21	0.8
	“I forgot to make an important phone call”	2	4	3	8	23	0.8
	“I told someone something that I did not mean to tell”	5	3	9	10	13	0.6
	“I forgot to pass on a message to someone”	2	2	2	8	26	0.9
	“I forgot to return a phone call”	3	1	6	10	20	0.8
	“I forgot to write an important letter”	5	3	4	7	21	0.7
	“I forgot to pay the bill when finishing a meal at a restaurant”	4	3	1	10	22	0.8
	“I forgot what I wanted to say in the middle of a sentence”	1	2	3	10	24	0.9
	“I forgot to say something important I had in mind at the beginning of a conversation”	1	2	3	12	22	0.9
	“I dialled someone on the phone and forgot who I had called by the time they answered”	3	5	3	6	23	0.7
	“I started writing a note or letter and forgot what I wanted to say”	3	6	1	9	21	0.8
	“I started to write a check and forgot to whom it was to be paid”	4	3	2	7	24	0.8

Caption: Items with i-CVI values greater than 0.8 are shaded in gray. PMQ = Prospective Memory Questionnaire; CAPM = Comprehensive Assessment of Prospective Memory. Appropriateness rating: ‘1’ = ‘Not appropriate’; ‘2’ = ‘Least appropriate’; ‘3’ = ‘Can’t say’; ‘4’ = ‘Somewhat appropriate’; ‘5’ = ‘Highly appropriate’.

Out of the 28 items, 21 with an i-CVI value above 0.8 were selected for further analysis. To ensure content uniqueness, researchers (DD, GB, HK, SM, and JB) meticulously scrutinized these 21 items for similarities during a joint session. Consensus among at least four of the five researchers was necessary to determine whether to retain or eliminate items before proceeding with further analysis. Accordingly, referring to Table 2, item 8 was excluded due to its similarity with item 24, both of which address forgetting content mid-conversation. Likewise, items 18 and 21 were deemed redundant with item 6, which covered forgetting to make a phone call, resulting in their removal. Item 5 remained, as it differed from item 20 in addressing forgetting to pass a message. Concerning bill payments, item 9 more aptly represented PM failures than item 23, hence its retention. Additionally, item 28, encompassing forgetting task specifics while performing it, was retained over item 27. This process yielded a final set of 14 items for the exploratory factor analysis.

Using the principal component matrix and varimax rotation, exploratory factor analysis was carried out using IBM SPSS version 25. A 0.50 minimum factor-loading threshold was set. To establish appropriate explanatory levels, the communality of the scale, which depicts the degree of variance in each dimension, was evaluated.

None of the items subjected to factor analysis was removed because all items had a factor loading of more than 0.50. The Kaiser – Meyer – Olkin MSA was 0.777, indicating an average degree of overlap, which requires further move on to factor analysis. Bartlett’s Test of Sphericity was measured to weigh the overall significance of the correlation matrix. The significant correlations between the correlation matrix and some of its components were statistically quantified using this measure. Bartlett’s Test of Sphericity showed significance, x^2^(n = 40) = 418.397 p < 0.001. The required value of 0.5 was exceeded by all communalities, indicating its suitability for the factor analysis.

Four factors were determined by factor analysis using a principal component matrix with varimax rotation, with all items having eigenvalues above 1. A four-dimensional structure was obtained as a result of this analysis. The four dimensions explained 79.45% of the variance in study items. Factor 1 included three items from the PMQ and one item from the CAPM questionnaire. Factor 2 included four items from the CAPM questionnaire. Factor 3 included two items from the CAPM questionnaire and one item from the PRMQ. Factor 4 included two from the PRMQ and one item from the CAPM. Based on the similarity of the items under each of the four factors and the nature of PM failure depicted through them, these factors were jointly labelled by the authors of the study and have been depicted along with the respective factor loadings in Table 3.

**Table 3:** Item numbers, corresponding questionnaires, and factor loadings from the factor analysis

Questionnaire	Items	Factor loading
Factor 1: PM failure due to loss of communicative content		
PMQ	“I started writing a note or letter and forgot what I wanted to say”	.848
PMQ	“I forgot to say something important I had in mind at the beginning of a conversation”	.843
PMQ	“I forgot what I wanted to say in the middle of a sentence”	.815
CAPM	“Arriving at a shop and forgetting what you planned to buy”	.744
Factor 2: PM failure due to loss of communicative intent		
CAPM	“Forgetting to make a telephone call you intended to make”	.537
CAPM	“Not remembering to pay bills”	.845
CAPM	“Forgetting to meet a friend at the pre-arranged time”	.756
CAPM	“Forgetting to pass on a message”	.610
Factor 3: PM cost due to ongoing interference		
CAPM	“If I am engrossed in another task, I find it difficult to remember to do things”	.904
CAPM	“Sometimes even though I remember that something has to be done, I forget to do it if I am interrupted (e.g., by a telephone call or by a person)”	.727
PRMQ	“Do you decide to do something in a few minutes time and then forget to do it?”	.675
Factor 4: PM failure linked to the priority of the communicative intent		
PRMQ	“Do you forget to tell someone something they had meant to mention a few minutes ago?”	.884
PRMQ	“Do you fail to mention or give something to a visitor that you were asked to pass on?”	.730
CAPM	“I frequently forget to do things that other people have asked me to do”	.620

Caption: PMQ = “Prospective Memory Questionnaire”; CAPM = “Comprehensive Assessment of Prospective Memory”; PRMQ = “Prospective and Retrospective Memory Questionnaire”

The first factor was labelled as PM failure due to loss of communicative content, the second factor as PM failure due to loss of communicative intent, the third factor as PM cost due to ongoing interference, and the last factor as PM failure linked to the priority of the intention to communicate.

## DISCUSSION

This study was conducted with the purpose of systematically identifying communication-related PM functions. Based on the appropriateness ratings provided by experts, 14 items from the three questionnaires were found to be important for communication. Factor analysis of these 14 items resulted in the extraction of four factors which could aid in classifying these items. Based on the commonality shared by the items in each factor and the construct they might indicate, these four factors were labelled. The four factors indicating the nature of PM failure were named PM failure due to loss of communicative content, PM failure due to loss of communicative intent, PM cost due to ongoing interference, and PM failure linked to the priority of communicative intent. The nature of the PM task and the nature of cognitive processes such as attentional capacity and executive control contributing to the PM task, compromise in PM performance due to the nature of the ongoing task^(21)^, and personal relevance of the PM task^(22)^ are frequently necessary for successful PM execution. The four factors obtained in the present study are discussed below with reference to PM properties and their possible association with communicative functions.

### PM failure due to ‘loss of content’

In this extracted factor, the items appraise PM failures as a result of loss of content, and could help clinicians identify comparable barriers related to communication. According to the nature of the PM task, this attribute is connected to the PM failure, i.e., “loss of content” wherein one might know that he or she needs to do or say something but might forget what had to be done or conveyed. In such PM failures, the intent to perform the task may not be affected; however, details of the intended task may be forgotten. For example, remembering visiting a general store but forgetting what one planned to buy (a typical scenario in an Indian context, where most customers ask shopkeepers for the planned items as compared to picking up goods from the shelves of a store). Such content-related deficiencies may be linked to age-related decreases in processing speed and executive functioning^(23)^. The fundamental tenet of communication is to exchange information (content) for cooperative building of meaning and sense. At the sender’s end, in a given social situation, if the message to be conveyed to an acquaintance is forgotten, they may find themselves in an uneasy situation. Similarly, if an intended message is not delivered, there may be an interruption in the flow of events at work or in personal life because of the failure to deliver the message.

### PM failure due to ‘loss of intent’

In this factor, the PM items might inform us about certain triggers linked to one’s intent to communicate, and therefore might be essential for an individual to implement communication-related intentions in everyday life; failure to do so might result in an intent-related deficit. The PM deficits linked to “loss of intent” are connected to cognitive functions, including attention and executive control, wherein one might not execute the intention of the task as a whole, such as ignoring a scheduled meeting with a friend. Remembering and performing an intent depends on the ability of a person to detect an event, interpret it as a cue, and execute the intention accordingly. Therefore, attentional capacities and executive mechanisms^(24)^ that encourage recall of self-initiated intentions at the correct moment may be limited when one is unable to recognize such a cue.

The ability to accurately detect a cue for PM retrieval depends on the focality of the PM cue, number of PM intentions, and complexity of the PM task^(25)^. The degree to which the PM cue and the ongoing task overlap determines the focality of the cue. Cues that are nonfocal in nature (with a low degree of overlap) are more difficult to detect than focal cues (with a high degree of overlap). In addition, the intricacy of the PM task and the number of intentions affect how easily the PM cue can be detected to recollect the PM intent. This affects the level of executive control necessary to effectively encode and recover intents, thereby altering performance on the PM task. Thus, the lack of triggers to activate intentions may explain why intent-related errors can occur. Such intentions are a significant element of socialization, and a person's integrity can be placed under the scanner at a societal level when intent-related errors occur frequently in environments such as home, work, and within the community.

### PM cost due to ongoing interference

The PM items extracted under the third factor could provide clinicians with insight into how typical activities in our daily routine (cognitive load) could prevent us from carrying out a PM task and assessing the mechanism of PM cost due to ongoing cognitive load. The first two items (“If I am engrossed in another task, I find it difficult to remember to do things”; “Sometimes even though I remember that something has to be done, I forget to do it if I am interrupted”) in this component provide information on the PM cost associated with a shift in attention allocation due to an ongoing task, whereas the third item (“Do you decide to do something in a few minutes time and then forget to do it”) aids in our comprehension of the PM costs associated with the time lag between task encoding and retrieval.

The third attribute, “PM cost due to ongoing interference,” deals with the compromise that occurs in PM performance owing to the nature of ongoing tasks. The demand for an ongoing task can lead to PM failure and result in higher PM costs. For example, getting engrossed in responding to e-mails at work, or forgetting to conduct scheduled lectures. Although most intentions are embedded in our ongoing daily routine, certain routines can tax more on cognitive capacity, resulting in PM-related communication issues. The concept of cost can be explained by reviewing the two PM monitoring theories. The first theory, preparatory attentional monitoring theory^(26)^, explains that to detect the presence of PM execution cues, attentional resources must be devoted to the task, while the second theory, two-process theory^(27)^, explains the need for constant target-checking behavior. Only through continuous monitoring in anticipation of PM targets is the chance of PM execution increased, thereby lowering PM costs. In addition to monitoring strategies, the overlap between ongoing and PM tasks (focality) is also important. According to the multiprocessing theory^(26)^, most focal tasks are retrieved spontaneously. Tasks with high focality, that is, if the nature of the PM task and the nature of the ongoing task are similar, have a higher probability of performing PM than tasks with low focality (nonfocal). However, tasks that are nonfocal in nature would require frequent monitoring. The delay between PM encoding and PM performance also plays a role. A longer delay would have greater PM costs than a shorter delay^(26)^.

### PM failure linked to priority of the communicative intent

The fourth attribute, ‘PM failure linked to priority of the intention to communicate’ addressed the relevance of the PM task on the personal level. Items under this attribute could be useful to clinicians for assessing PM failures that have unfavorable effects on someone else. This attribute highlights the importance that an individual assigns to the intent of a task based on the level of importance that a particular task holds. Quite often in our daily lives, as a result of socialization, we might be asked by a family member, colleague, or acquaintance to perform tasks in the future. However, if one does not consider it important, and there is a lack of intrinsic motivation, one might forget to carry it out. The foundation for such an intention's significance also depends on the participants’ values, ambitions, goals, social motives (giving participants instructions that an intention is crucial for someone else), and anticipated effects^(22)^. One assigns priority to a PM activity based on whether he/she receives a benefit from it (reward) or whether the task is appealing as a whole (attractive). When communicating an intention, highlighting the significance of a PM task may lead to higher levels of intention activation, enhanced sensitivity to potential PM target events, and improved accessibility to potential tasks. In our everyday lives, we meet multiple people, and requests might be made to us to perform communication-related acts to network and build relationships at home or in society. Therefore, forgetting PM tasks because of the priority assigned to communicative intent could result in communication disadvantages for the individual and his or her social circle.

From a neurobiological standpoint, specific brain regions, including the prefrontal cortex, medial frontal lobe, medial temporal lobe, posterior parietal areas, hippocampal area, and subthalamic nucleus, have been identified to play crucial roles across various stages of PM, such as intention formation, retention, initiation, and execution^(28)^. Notably, these structures are also integral to communicative functions^(29)^. For example, the prefrontal cortex is responsible for integrating sensory information to finely regulate behavior and decision making during social interactions. Similarly, the medial temporal lobe significantly contributes to semantic processing in language comprehension and encodes the temporal organization of memories, which is a vital process for narrative skills in communication. Moreover, the hippocampus participates in shared representation and interpersonal predictive coding, thereby collaboratively enhancing communication at multiple levels. Hence, the considerable neurobiological overlap between regions facilitating PM function and communication appears relevant, particularly in emphasizing the role of PM abilities in diverse communicative functions, as highlighted in the present study.

While highlighting the significance of PM in everyday communication, the findings of the present study encourage SLP clinicians to broaden cognitive communicative assessments beyond measures related to retrospective memory. The inclusion of PM as a crucial aspect is essential. Furthermore, the communication-related PM elements and constructs proposed in this study could assist SLPs in devising innovative assessment tools such as questionnaires. These tools could complement performance-based PM assessments, aiding clinicians in identifying specific communication-related PM deficits in both healthy and pathologically aging populations. Understanding individual failures in communication-related PM across the four factors outlined in this study—'loss of content,' 'loss of intent,' 'cost due to ongoing interference,' and 'priority of communicative intent'—can guide clinicians in identifying the nature of PM deficits. This understanding can help plan tailored intervention strategies accordingly. For instance, strategies such as spaced retrieval and semantic associations could aid individuals encountering PM failures associated with communicative content and priority. Similarly, techniques such as visual imagery and implementation intention could assist those facing PM deficits associated with communicative intent and the costs of ongoing tasks.

### Limitations and future directions

The present study had certain limitations. It considered only three of the most widely used questionnaires to extract the communication-related aspects of PM. It is possible that there could be additional communication-related PM aspects in other self-rated PM questionnaires. As this study concentrated solely on items derived from the questionnaires, it did not provide an opportunity for SLP experts to suggest additional questions that could contribute to this research. Nonetheless, the methodological strength of this study lies in the systematic and expert-driven process of identifying communication-related aspects of PM from a few of the most widely used questionnaires. The identified communication-related aspects of PM could serve as a foundation for future researchers to create innovative self-assessment questionnaires aimed at evaluating communication-related PM skills among healthy and pathologically aging populations. While this study primarily focused on delineating communication-related PM functions among aging adults, these findings may also hold relevance for SLPs aiming to assess PM functions across various communication disorders and age demographics.

## CONCLUSION

Communication-associated PM remains relatively under-recognized in the existing literature. This study endeavoured to identify PM attributes pertinent to communication, leveraging the expertise from professionals in the field. Following ratings from 40 experts specializing in communication disorders, this study identifies important PM attributes which could be linked to communication and introduces four key factors: 'PM failure due to loss of communicative content,' 'PM failure due to loss of communicative intent,' 'PM cost due to ongoing interference,' and 'PM failures associated with the priority of communicative intent.' This study advocates the incorporation of PM as a crucial assessment domain in cognitive-communication research. These highlighted factors are proposed as a framework for the development of tools aimed at assessing PM abilities. Also, these factors offer a guiding structure for PM intervention, facilitating the targeted resolution of specific challenges encountered in everyday communication among healthy and pathologically aging individuals.
